# Understanding lipidomics associations and the lipoprotein-related caveats in population epidemiology

**DOI:** 10.1093/aje/kwae445

**Published:** 2024-11-28

**Authors:** Siyu Zhao, Pauli Ohukainen, Johannes Kettunen, Marjo-Riitta Järvelin, Mika Kähönen, Terho Lehtimäki, Jorma Viikari, Olli T Raitakari, Ville-Petteri Mäkinen, Mika Ala-Korpela

**Affiliations:** Systems Epidemiology, Faculty of Medicine, University of Oulu, 90014 Oulu, Finland; Research Unit of Population Health, Faculty of Medicine, University of Oulu, 90014 Oulu, Finland; Biocenter Oulu, University of Oulu, 90014 Oulu, Finland; Systems Epidemiology, Faculty of Medicine, University of Oulu, 90014 Oulu, Finland; Research Unit of Population Health, Faculty of Medicine, University of Oulu, 90014 Oulu, Finland; Biocenter Oulu, University of Oulu, 90014 Oulu, Finland; Systems Epidemiology, Faculty of Medicine, University of Oulu, 90014 Oulu, Finland; Research Unit of Population Health, Faculty of Medicine, University of Oulu, 90014 Oulu, Finland; Biocenter Oulu, University of Oulu, 90014 Oulu, Finland; Department of Public Health and Welfare, Finnish Institute for Health and Welfare, 00271 Helsinki, Finland; Research Unit of Population Health, Faculty of Medicine, University of Oulu, 90014 Oulu, Finland; Department of Epidemiology and Biostatistics, MRC Centre for Environment and Health, School of Public Health, Imperial College London, London W12 0BZ, United Kingdom; Department of Life Sciences, College of Health and Life Sciences, Brunel University London, London UB8 3PH, United Kingdom; Department of Clinical Physiology, Tampere University Hospital, and Finnish Cardiovascular Research Center Tampere, Faculty of Medicine and Health Technology, Tampere University, 33270 Tampere, Finland; Department of Clinical Chemistry, Fimlab Laboratories, and Finnish Cardiovascular Research Center Tampere, Faculty of Medicine and Health Technology, Tampere University, 33270 Tampere, Finland; Department of Medicine, University of Turku, 20014 Turku, Finland; Division of Medicine, Turku University Hospital, 20014 Turku, Finland; Division of Medicine, Turku University Hospital, 20014 Turku, Finland; Research Centre of Applied and Preventive Cardiovascular Medicine, University of Turku, 20014 Turku, Finland; Centre for Population Health Research, University of Turku and Turku University Hospital, 20014 Turku, Finland; Department of Clinical Physiology and Nuclear Medicine, Turku University Hospital, 20014 Turku, Finland; Systems Epidemiology, Faculty of Medicine, University of Oulu, 90014 Oulu, Finland; Research Unit of Population Health, Faculty of Medicine, University of Oulu, 90014 Oulu, Finland; Biocenter Oulu, University of Oulu, 90014 Oulu, Finland; Systems Epidemiology, Faculty of Medicine, University of Oulu, 90014 Oulu, Finland; Research Unit of Population Health, Faculty of Medicine, University of Oulu, 90014 Oulu, Finland; Biocenter Oulu, University of Oulu, 90014 Oulu, Finland; NMR Metabolomics Laboratory, School of Pharmacy, Faculty of Health Sciences, University of Eastern Finland, 70210 Kuopio, Finland

**Keywords:** bias, clinical lipids, confounding, lipidomics, lipoproteins, mediation, overadjustment, triglycerides

## Abstract

Mass spectrometry lipidomics is becoming customary to analyze serum/plasma samples in epidemiology. The measurables are molecular constituents of lipoprotein particles, but very little is known about the consequences of adjusting lipidomics data with lipoprotein measures. We studied 2 population cohorts with 5657 and 2036 participants. Liquid chromatography/tandem mass spectrometry lipidomics was applied to analyze 24 molecular lipid classes and nuclear magnetic resonance spectroscopy to quantify 7 lipoprotein lipids plus apolipoprotein A-I and B. The associations of these measures were analyzed via partial Spearman’s correlations. The effects of 9 different lipoprotein adjustments on these interrelationships were assessed. Multivariable regression modeling with these adjustments was also performed for the associations between the lipidomics data and body mass index. These novel large-scale lipidomics data and their associations between the lipoprotein measures were coherent in both population cohorts, confirming the compatibility of the analytical approaches. Simulated data were generated to corroborate the mediation effects. The lipoprotein-related lipid transport and metabolism inherently mediate the lipidomics associations, as evident from the striking effects of the lipoprotein adjustments. These effects and their relevance to the interpretations of lipidomics data are presented and discussed in detail for the first time. The combined lipoprotein lipid adjustments appear prone to overadjustment and arbitrary biases.

## Key messages:

Here we present a detailed assessment of the fundamental molecular framework of circulatory lipoproteins (nuclear magnetic resonance spectroscopy) and their lipid constituents (liquid chromatography/tandem mass spectrometry [LC-MS/MS]) and how various adjustments with lipoprotein-related measures affect associations and epidemiologic regression models and their interpretations with LC-MS/MS lipidomics data. The lipoprotein-centered lipid transport system inherently intertwines the lipidomics data with the lipoprotein measures in a complex, nonlinear way, and thereby the combined lipoprotein lipid adjustments (triglycerides, cholesterol or low-density lipoprotein cholesterol, and high-density lipoprotein cholesterol) of regression models with LC-MS/MS lipidomics data are very difficult to interpret and prone to bias. Associations of the lipoprotein lipid constituents, analyzed by LC-MS/MS lipidomics and adjusted with combined lipoprotein measures, should be interpreted with extreme care due to a high likelihood for biased results; instead, it would be more prudent to adjust for individual lipoprotein measures—triglycerides, various cholesterol measures, and apolipoprotein B—and stemming the interpretations from their known compositional and metabolic roles.

## Introduction

Lipoproteins are heterogeneous noncovalent protein-lipid particles with key functions in circulatory lipid transport and metabolism. In serum/plasma samples separated from blood collections, they are basically the sole vehicles for all lipid molecules,^1^^-^^5^ although albumin contains the bulk of free fatty acids^6^ and transports also some lysophosphatidylcholines^7^^,^^8^ and likely other lyso-types of lipid molecules with 1 fatty acid chain.

Lipoprotein-related lipid measures have played a key role in the risk assessment of cardiometabolic conditions since the 1950s.^9^ Circulating triglycerides (TG), total cholesterol (TC), and high-density lipoprotein cholesterol (HDL-C) have been the routine panel of measurements for decades, with TC more recently often replaced by low-density lipoprotein cholesterol (LDL-C). In addition, improved analytical techniques have shaped the contemporary convention toward a more comprehensive measurement panel in assessing cardiometabolic risk. The additional measures typically include separate assessment of very low-density and intermediate-density lipoprotein cholesterol (VLDL-C and IDL-C, respectively), as well as apolipoprotein A-I (apoA-I) and B (apoB).^10^^,^^11^ Remnant cholesterol, defined as (non-LDL, non-HDL)-C, is also often nowadays considered valuable for risk assessments.^12^^-^^14^ Findings from recent genetic epidemiology analyses with Mendelian randomization^15^ have been transformative, indicating that all the abovementioned lipoprotein-related measures would be independently, and causally, related to coronary heart disease (although the causal role of HLD-C and apoA-I is still ambiguous^11^^,^^16^^,^^17^) and many of them also for multiple other diseases, for example, stroke and Alzheimer disease.^11^

**Figure 1:**
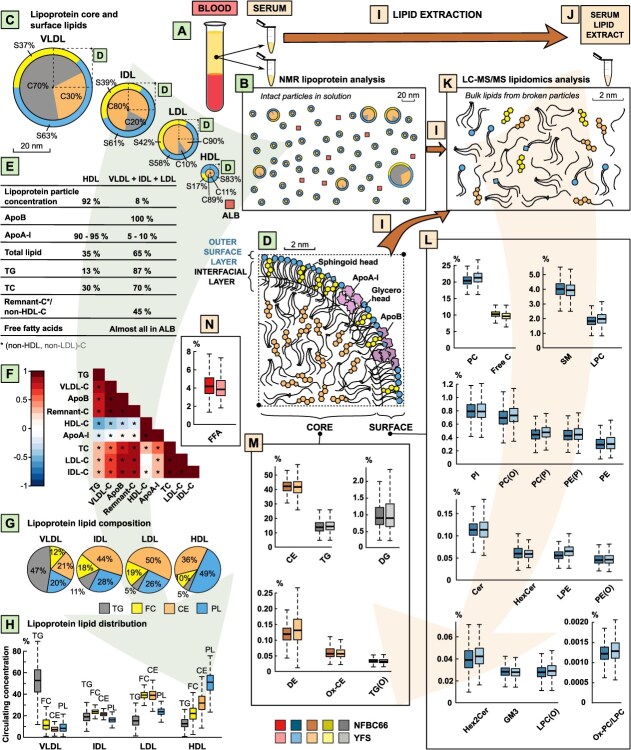
An overall illustration of the fundamental factors inherently linking the serum samples to lipoprotein and lipidomics data. (A) Blood serum samples are analyzed as such in NMR spectroscopy (B), but the LC-MS/MS lipidomics analyses (K) call for a lipid extraction process (I) before experimentation. (B-D) In intact serum, lipid molecules reside within a complex mixture of lipoprotein particles (and free fatty acids, some lysophosphatidylcholines, and some other lyso-type of lipid molecules with albumin). (E-H) Lipoprotein particles can be characterized by different subfractions, each of which has a typical lipid content and associations with other subfractions and lipid measures. (I) Lipid extraction is a key process to make serum samples analyzable by LC-MS/MS lipidomics (K). This results in a sample that consists of all the individual lipid molecules in the original serum sample as a homogeneous lipid-soluble mixture with all the lipoprotein particles broken and free fatty acids as well as the lyso-type of lipid molecules dissolved from albumin (J); all the information on the specific lipoprotein origin of the individual lipid molecules is lost (K). However, LC-MS/MS lipidomics leads to very rich and detailed analyses on serum lipids. (L-N) Unique results are illustrated for the 24 most abundant molecular lipid classes in 2 independent large population studies: the Northern Finland Birth Cohort 1966 (NFBC66) with 5657 participants and the Cardiovascular Risk in Young Finns Study (YFS) with 2036 participants. The lipid class data are shown as the percentage of total lipids (mol%) in serum. The boxplots represent medians with interquartile ranges and the minimum (Q1 – 1.5 * IQR) and maximum (Q3 + 1.5 * IQR) values with potential outliers. The absolute concentration data for the lipoprotein measures are given in Table 1 and for the lipidomics measures in Table 2. Please note the different size scale in (B), (D), and (K). Abbreviations: VLDL, very-low-density lipoprotein; IDL, intermediate-density lipoprotein; LDL, low-density lipoprotein; HDL, high-density lipoprotein; ALB, albumin; NMR, nuclear magnetic resonance; LC-MS/MS, Liquid chromatography-mass spectrometry; ApoA-I, apolipoprotein A-I; ApoB, apolipoprotein B; TG, triglycerides; C, cholesterol; FC, free cholesterol; CE, cholesteryl esters; PL, phospholipids; PC, phosphatidylcholines; SM, sphingomyelins; LPC, lysophosphatidylcholines; PI, phosphatidylinositols; PC(O), alkylphosphatidylcholines; PC(P), alkenylphosphatidylcholines; PE(P), alkenylphosphatidylethanolamines; PE, phosphatidylethanolamines; Cer(d), ceramides; HexCer, monohexosylceramides; LPE, lysophosphatidylethanolamines; PE(O), alkylphosphatidylethanolamines; Hex2Cer, dihexosylceramides; GM3, GM3 gangliosides; LPC(O), lysoalkylphosphatidylcholines; Ox-PC/LPC, oxidised phosphatidylcholines/lysophosphatidylcholines; DG, diacylglycerols; DE, dehydrocholesterol esters; Ox-CE, oxidised cholesteryl esters; TG(O), alkyldiacylglycerols; FFA, free fatty acids.

The above background is of the essence regarding lipidomics studies of serum/plasma samples. Unlike enzymatic lipid assays or nuclear magnetic resonance (NMR) spectroscopy, mass spectrometry (MS) lipidomics calls for lipid extraction of the serum/plasma samples as an integral part of the analyses, as illustrated in Figure 1.^18^^-^^20^ Thus, the preparations analyzed by MS lipidomics represent pooled mixtures of all the various lipid molecules from all the circulating lipoprotein particles. All the information from the specific lipoprotein origin of a certain lipid in the circulation is lost in the extraction phase. This calls attention to the integration of lipoprotein data when interpreting serum/plasma lipidomics associations. However, this fact has not been addressed in detail in the literature. Conversely, it is often either disregarded or dealt with via adjusting analyses in a straightforward manner for TG + TC + HDL-C, implicitly giving the rationale to consider the results “independent of standard clinical lipids.”^21^^-^^24^

**Table 1:** Clinical characteristics and lipoprotein measures in NFBC66 and YFS.

**Clinical characteristics**	**NFBC66**	**YFS**
Number of participants	5657	2036
Number of females (%)	3168 (56.0%)	1109 (54.5%)
Age (y)	46.6 [46.2-47.1]	43.0 [37.0-46.0]
BMI (kg/m^2^)	26.1 [23.5-29.3]	25.8 [23.1-29.1]
**Lipoprotein lipids (mmol/L)**		
Total triglycerides Total cholesterol	1.13 [0.83-1.61] 5.62 [4.97-6.35]	1.09 [0.79-1.58] 5.25 [4.60-5.98]
VLDL cholesterol	0.80 [0.63-1.02]	0.70 [0.55-0.92]
IDL cholesterol	0.92 [0.78-1.08]	0.84 [0.71-0.99]
LDL cholesterol	2.18 [1.81-2.63]	2.00 [1.63-2.41]
Remnant cholesterol	1.74 [1.45-2.09]	1.57 [1.29-1.89]
HDL cholesterol	1.63 [1.36-1.92]	1.61 [1.35-1.92]
**Apolipoproteins (g/L)**		
Apolipoprotein B Apolipoprotein A-I	1.03 [0.88-1.21] 1.73 [1.59-1.89]	1.00 [0.82-1.13] 1.67 [1.54-1.84]

Abbreviations: BMI, body mass index; HDL, high-density lipoprotein; IDL, intermediate-density lipoprotein; LDL, low-density lipoprotein; VLDL, very low-density lipoprotein.

The values are median [Q1-Q3]. Remnant cholesterol refers to (non-LDL, non-HDL) cholesterol.

Confounding, mediation, and overadjustment biases are common sources of erroneous conclusions in the epidemiology of pleiotropic traits.^25^ Combined lipidomics and lipoprotein data are likely to be particularly adversely affected due to the tight biophysical and metabolic relationships that lead to large sets of highly collinear measures.^5^^,^^26^ In this work, we present novel data from 2 independent population cohorts with 7693 participants with serum samples, analyzed by NMR spectroscopy for an extensive 9-measure clinical lipoprotein panel and by liquid chromatography/tandem mass spectrometry (LC-MS/MS) lipidomics for 24 molecular lipid classes. We investigate, for the first time in detail, how various individual and combined adjustments with lipoprotein measures affect the results and their interpretation with lipidomics data.

## Methods

### Population cohorts

Two independent population cohorts were studied cross-sectionally: the Northern Finland Birth Cohort 1966 (NFBC66)^27^ with 5657 participants (median age 46 years, 56% women) and the Cardiovascular Risk in Young Finns Study (YFS)^28^ with 2036 participants (median age 43 years, 55% women). More details on these cohorts are given in the supplementary material. NFBC66 is one of the biggest epidemiologic cohorts with LC-MS/MS data available, and together, these cohorts comprise a unique combination of large-scale epidemiologic data for comprehensive lipoprotein panels and lipidomics measures.

### NMR spectroscopy analysis for the lipoprotein panel

We applied an NMR platform that has been widely used in epidemiology and genetic studies over the past 15 years and for which the general methodological issues have been published and discussed previously.^5^^,^^20^^,^^29^^-^^39^ We included a 9-measure lipoprotein panel consisting of key lipid measures for the 4 major lipoprotein fractions VLDL, IDL, LDL, and HDL as well as apoA-I and apoB, which are abundant in the circulation and are the 2 most analyzed apolipoproteins in epidemiologic studies.^11^  Figure 1E and F demonstrate the metabolic overlap and interrelationships of these lipoprotein measures, and the absolute concentration data in both cohorts are given in Table 1. TG, free cholesterol (FC), cholesteryl ester (CE), and phospholipid (PL) concentration data from the NMR analysis were used to outline the lipid compositions of the 4 key lipoprotein fractions (Figure 1G) and their relative contributions to lipid transport in the bloodstream (Figure 1H). A more detailed dissection of the structural and metabolic relationships of comprehensive lipoprotein data can be found in our previous work.^5^

### LC-MS/MS mass spectrometry analysis for the molecular lipid classes

The LC-MS/MS experiments were performed at the Baker Heart and Diabetes Institute, Melbourne, Australia. Serum lipids were extracted as previously described^19^ and the molecular lipid classes analyzed using an adapted method as described earlier.^21^ Both NFBC66 and YFS were run on the same methodology, of which more details can be found in the supplementary material. We included the 24 most abundant molecular classes into the analyses to limit the results to those most pertinent to convey the key message in this work and to prevent the common overflow of (highly correlated) results and complicated visualizations with lipidomics data (Figure 1L-N). Data wise, this is a minor exclusion since these lipid classes constitute 99.91% and 99.90% of total serum lipid concentrations in NFBC66 and in YFS, respectively. The absolute concentration data for these 24 lipid classes in both cohorts are given in Table 2. The data for both cohorts were scaled according to a set of National Institute of Standards and Technology (NIST) plasma samples.

**Table 2:** LC-MS/MS lipidomics measures for the total concentrations of the 24 most abundant circulating lipid classes (μmol/L) in NFBC66 and YFS.

	**NFBC66**	**YFS**
Cluster 1 (mol %)	30.4%	30.7%
Oxidized phosphatidylcholine/ lysophosphatidylcholines [Ox-PC/LPC]	0.12 [0.10-0.14]	0.13 [0.11-0.16]
Oxidized cholesteryl esters [Ox-CE]	5.45 [4.27-7.26]	5.62 [4.48-7.02]
Phosphatidylcholines [PC]	1901.3 [1667.4-2133.3]	2062.7 [1853.7-2286.8]
Free cholesterol [Free C]	933.8 [828.4-1044.6]	906.9 [793.5-1034.9]
Ceramides [Cer]	9.95 [8.21-12.0]	10.4 [8.53-12.5]
Cluster 2 (mol %)	1.1%	1.2%
Phosphatidylinositols [PI]	73.1 [61.1-87.3]	76.7 [64.8-90.7]
Phosphatidylethanolamines [PE]	26.1 [19.7-35.2]	28.2 [20.6-39.6]
Alkyldiacylglycerols [TG(O)]	3.12 [2.69-3.65]	3.14 [2.69-3.69]
Cluster 3 (mol %)	15.1%	15.6%
Diacylglycerols [DG]	88.7 [66.7-124.3]	92.9 [65.9-136.6]
Triacylglycerols [TG]	1233 [938.6-1624]	1337 [1076.2-1664.9]
Cluster 4 (mol %)	1.8%	1.9%
Alkenylphosphatidylethanolamines [PE(P)]	39.4 [32.2-47.8]	42.6 [34.2-52.8]
Alkylphosphatidylethanolamines [PE(O)]	3.59 [2.86-4.56]	3.83 [2.95-4.92]
Alkylphosphatidylcholines [PC(O)]	63.9 [54.1-75.2]	70.9 [59.5-83.8]
GM3 gangliosides [GM3]	2.58 [2.16-3.05]	2.66 [2.26-3.05]
Alkenylphosphatidylcholines [PC(P)]	40.5 [33.3-49.0]	46.0 [37.3-54.9]
Dehydrocholesterol esters [DE]	11.0 [9.18-13.1]	12.6 [9.47-16.1]
Cluster 5 (mol %)	1.9%	2.0%
Lysophosphatidylcholines [LPC]	166.4 [136.9-200.6]	189.6 [157.2-222.2]
Lysophosphatidylethanolamines [LPE]	4.53 [3.76-5.48]	5.71 [4.63-6.87]
Cluster 6 (mol %)	0.1%	0.1%
Dihexosylceramides [Hex2Cer]	3.54 [2.88-4.35]	4.04 [3.31-4.91]
Monohexosylceramides [HexCer]	4.91 [4.06-5.92]	5.03 [4.22-5.97]
Lysoalkylphosphatidylcholines [LPC(O)]	2.51 [2.1-2.96]	2.78 [2.30-3.28]
Cluster 7 (mol %)	4.4%	4.1%
Free fatty acids [FFA]	389 [316.5-479.8]	372.9 [300.4-473.5]
Cluster 8 (mol %)	45.2%	44.4%
Sphingomyelins [SM]	372.5 [317.9-428.5]	382.6 [330.5-432.5]
Cholesteryl esters [CE]	3861 [3424-4330]	3932 [3479-4393]

**Figure 2:**
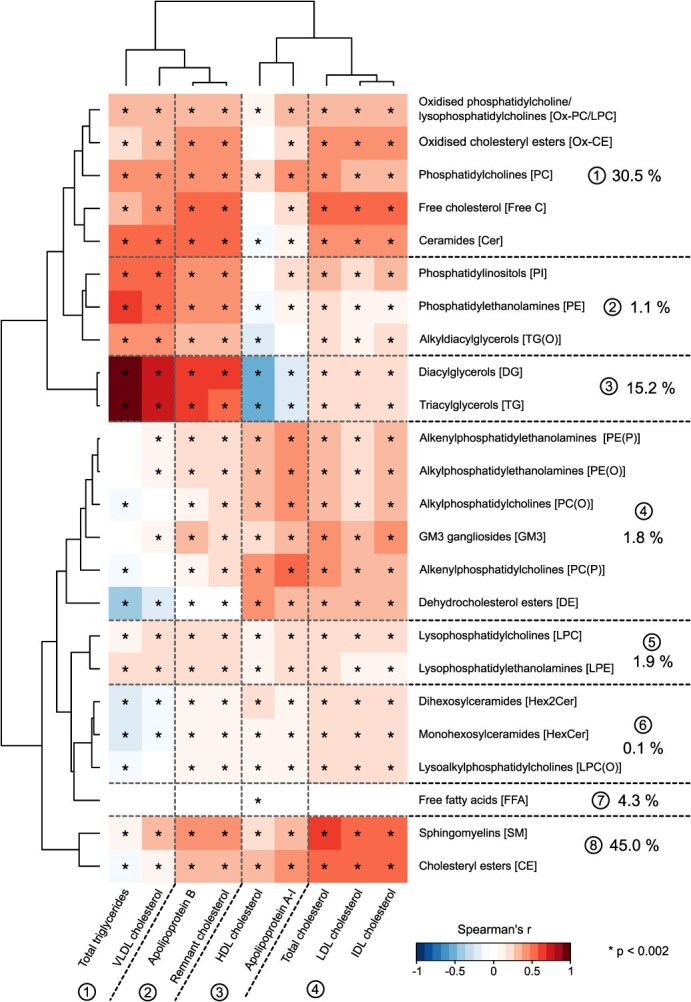
Associations of the 9 key lipoprotein measures with the 24 most abundant lipidomics lipid classes as indicated by partial Spearman’s rank correlations. The data are from 2 independent large population studies, NFBC66 with 5657 participants and YFS with 2036 participants. All correlations were adjusted for sex in both cohorts and age in YFS. Both cohorts were analyzed separately and then meta-analyzed. The 2-dimensional hierarchical clustering is based on the meta-analyzed results for both cohorts, and the resulting ordering is preserved in all the following heatmaps in Figure 3. The general association characteristics are summarized via 4 and 8 metabolic clusters for the lipoprotein and lipidomics data, respectively. The percentages shown for the lipidomics clusters depict the contribution of each cluster to the circulating total lipid concentration. *P* < .002 is marked with an asterisk in the map to indicate a multiple testing corrected association. The abbreviations are as explained in the caption for Figure 1.

The clusters refer to Figure 2. The values are median [Q1-Q3].

### Statistical analyses

**Figure 3:**
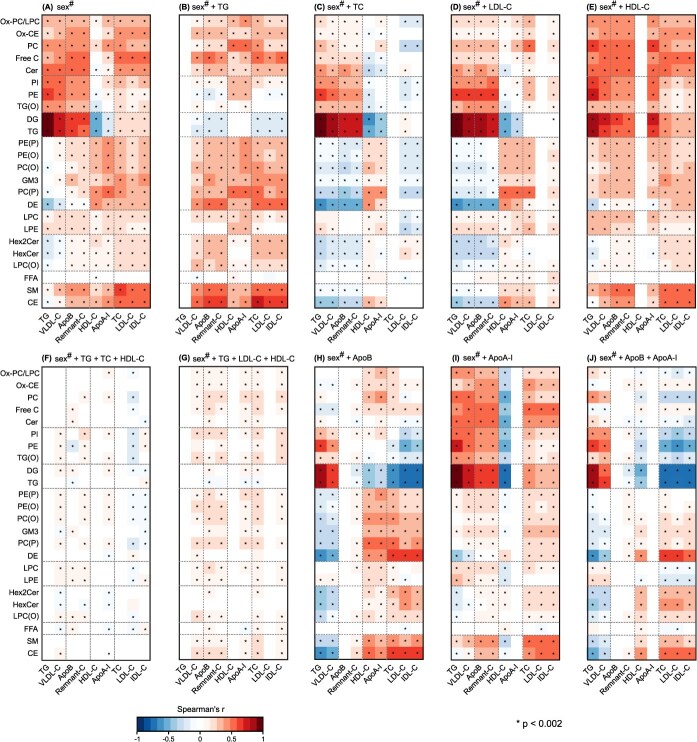
Associations of the 9 key lipoprotein measures with the 24 most abundant lipidomics lipid classes adjusted for different lipoprotein measures and their combinations. The data are from 2 independent large population studies, NFBC66 with 5657 participants and YFS with 2036 participants. The lipoprotein adjustments in (B-J) are in addition to the adjustment for sex in both cohorts and age in YFS (marked as sex^#^) (A). Both cohorts were analyzed separately and then meta-analyzed. All correlations are partial Spearman’s rank correlations, and the organization of the heatmaps is from Figure 2. *P* < .002 is marked with an asterisk in the maps to indicate a multiple testing corrected association. The abbreviations are as explained in the caption for Figure 1.

Partial Spearman’s correlations were calculated between all the individual measures in the lipoprotein panel (Figure 1F) and between all the lipoprotein and lipidomics measures (Figure 2). Both cohorts were analyzed separately and then combined via inverse variance weighted meta-analysis. All correlations were adjusted for sex in both cohorts and age in YFS. The color-coded heatmap in Figure 2 was hierarchically clustered in both dimensions and the resulting order of the measures preserved in the following analyses with additional adjustments for the key lipoprotein measures and their combinations: TG, TC, LDL-C, HDL-C, TG + TC + HDL-C, TG + LDL-C + HDL-C, apoB, apoA-I, and apoB + apoA-I (Figure 3). Results for the individual cohorts are shown in Figures S1 to S3.

**Figure 4:**
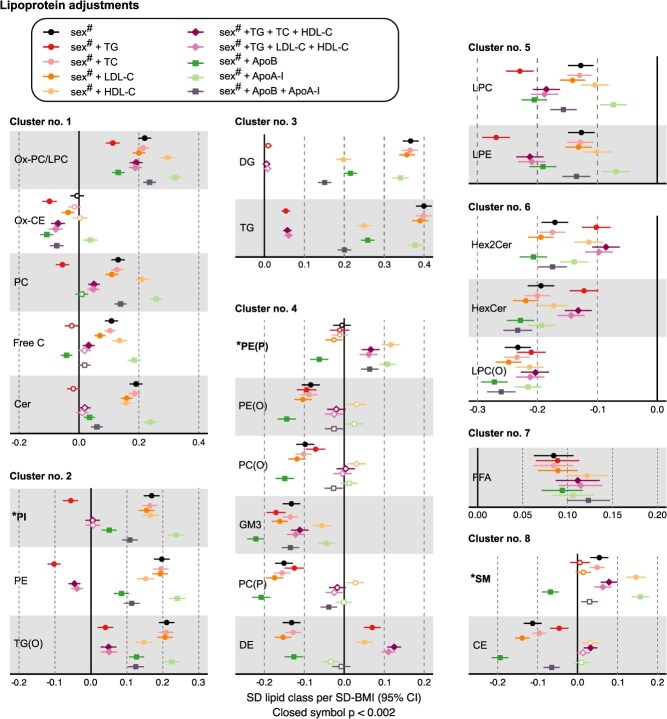
Illustration of the effects of various lipoprotein adjustments on the associations of the 24 most abundant lipidomics lipid classes with BMI via linear regression analyses. The data are from 2 independent large population studies, NFBC66 with 5657 participants and YFS with 2036 participants. Both cohorts were analyzed separately and then meta-analyzed. Association magnitudes are reported in standard deviation (SD) units throughout to ease the comparison across multiple measures with markedly varying absolute concentrations (Table 2). Sex^#^ refers to adjusting for sex in both cohorts and age in YFS. *P* < .002 is marked with an asterisk in the maps to indicate a multiple testing corrected association. The results for the emphasized lipid classes, namely **PI**, **PE(P)**, and **SM**, are discussed in more detail in the text. The abbreviations are as explained in the caption for Figure 1.

The effects of these lipoprotein adjustments were exemplified for the associations of the 24 lipidomics measures with body mass index (BMI) via linear regression analyses (Figure 4). As for the correlations, both cohorts were analyzed separately and then combined via inverse variance weighted meta-analysis (results for the individual cohorts are given in Figures S4 and S5). Association magnitudes are reported in standard deviation (SD) units throughout to ease the comparison across multiple measures with markedly varying absolute concentrations.

Twenty-five principal components explained over 95% of the variation in the 24 lipid classes and the 9 lipoprotein measures in both cohorts. Therefore, we set the 5% Bonferroni-adjusted type I error threshold at *P* < .05/25 = .002. Extreme values for both lipoprotein and lipidomics measures were truncated in all analyses to third quartile +8 × interquartile range. Extreme values were rare (Table S1) and had negligible effects on the results. All the analyses were done with the R software (version 4.2.1).

### Simulated data

**Figure 5:**
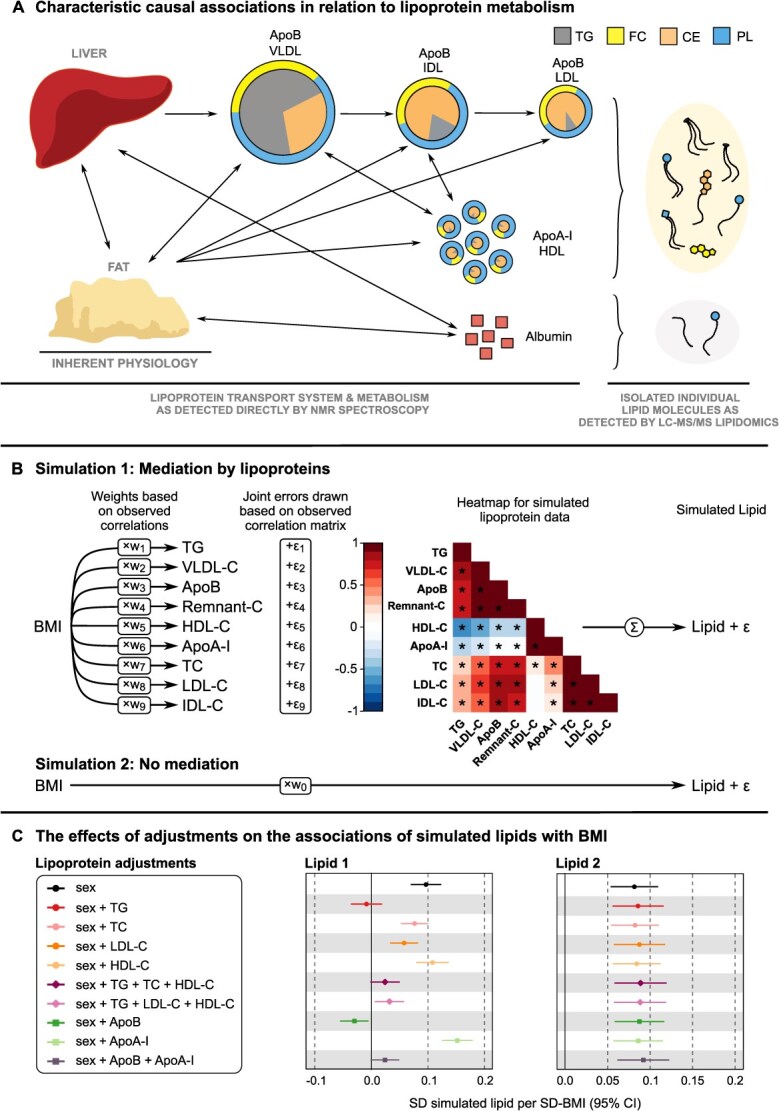
(A) Illustration of causal associations between various tissues and lipoprotein metabolism, as well as causal associations between various lipoprotein particles. The arrowheads represent a causal direction. This figure is a schematic representation and should not be interpreted as a formal directed acyclic graph. (B) The basis for simulated lipid data representing pure lipoprotein mediation (simulation 1 with no simulated direct association between lipid 1 and BMI) and no mediation by lipoprotein particles (simulation 2 with simulated direct positive association between lipid 2 and BMI). (C) The effects of various lipoprotein adjustments on the associations of the 2 simulated lipids with BMI via linear regression analyses.

To illustrate that lipoprotein mediation leads to the results observed with real data, 2 sets of simulated data were prepared and analyzed, as illustrated in Figure 5. First, we reproduced a biological system in which the circulating lipid (referring to LC-MS/MS data) is purely mediated by lipoprotein particles (referring to NMR data) (Figure 5B, simulation 1). This simulation model was set up to reproduce the observed pattern of correlation coefficients between BMI and lipoprotein measures, and it was used to simulate a lipid concentration for 5657 participants (as in NFBC66). The simulated lipoprotein mediation was expressed as a causality graph where BMI affects each lipoprotein measure with a specific strength (w_1_ to w_9_ in Figure 5B) and where the interactions between lipoprotein particles are modeled via mutually correlated random variation (ε_1_ to ε_9_). Both features were necessary to reproduce the observed correlation structure connecting BMI and lipoprotein measures. The simulation was implemented by drawing random values from a normal distribution and multiplying these values with w_1_ to w_9_ to create 9 lipoprotein variables with ε_1_ to ε_9_ added. The final lipid concentration was an unweighted sum of the 9 lipoprotein measures with measurement noise drawn from a normal distribution. In this simulation, we did not introduce any direct association between the lipid and BMI.

Second, we reproduced a biological system in which the circulating lipid is not associated with lipoprotein metabolism (Figure 5B, simulation 2). This was done by drawing a random value for BMI that was directly copied as the lipid concentration but with measurement noise added. In this simulation, we introduced a positive association between the lipid and BMI.

## Results and discussion

### Fundamental molecular context

Figure 1 illustrates the key factors inherently linking the blood serum samples to lipoprotein and lipidomics data. Lipoprotein analyses with NMR proceed directly with the serum sample without any particular sample pretreatments (Figure 1A and B).^29^^,^^30^ The increased use of NMR in lipoprotein analytics comes from the fact that the technology is directly sensitive to the lipoprotein particle size and can therefore lead to comprehensive quantitative lipid data on various lipoprotein particles (Figure 1C).^5^^,^^40^ The situation is radically different for MS lipidomics techniques. To achieve the precise molecular resolution, the individual molecular constituents must be directly available (ie, the serum lipoprotein structures) (Figure 1B-D), and heterogeneity in the original sample must be simplified. This is achieved by a lipid extraction procedure^19^ (marked I in multiple places in Figure 1), resulting in a sample that consists of all the individual lipid molecules in the original serum sample as a homogeneous lipid-soluble mixture (Figure 1J). This sample is used in the LC-MS/MS analysis with the inherent limitation that all the information on the specific lipoprotein origin of the individual lipid molecules is lost (Figure 1K).

Lipoprotein metabolism is naturally a continuum linking all apoB-containing lipoprotein particles metabolically together.^5^^,^^12^^,^^41^^,^^42^ This is illustrated in Figure 1F, which shows strong positive associations between TC and apoB with all the apoB-containing particle cholesterol measures (VLDL, IDL, LDL, and remnant). In addition, HDL metabolism is tightly interconnected with circulating TG,^1^^,^^5^^,^^16^^,^^43^^-^^45^ resulting in a strong negative correlation between TG and HDL-C (and apoA-I, the main apolipoprotein component in HDL particles). However, the lipoprotein particles per se are heterogeneous (eg, in their structure and lipid composition) (Figure 1C and G). Their contribution to the circulating lipids also varies markedly between the lipid classes; for examples, HDL particles transport only 13% of triglycerides in the bloodstream, while apoB-containing particles transport 87%, of which over 60% resides in VLDL (Figure 1E and H). These convoluted metabolic and structural issues^5^ are the fundamental source for the perplexity in understanding adjustment effects with lipoprotein measures.

The presented absolute concentration data for the 24 molecular lipid classes in 2 independent large population studies are unique (Figure 1L-N and Table 2). They are in good agreement with the current view on the relative concentrations of plasma lipids.^46^ The coherence of the results for the 2 independent epidemiologic cohorts is striking. While similarity for the lipidomics data is expected due to the close correspondence of the lipoprotein profiles in both cohorts (Table 1 and Figure S1), this is a pioneering demonstration of the quantitative performance of LC-MS/MS analyses^21^^-^^23^^,^^47^ in large-scale epidemiology.

NMR spectroscopy data on lipoprotein lipids are limited to 4 major lipid categories: TG, FC, CE, and PL. However, this information is available for various lipoprotein categories informing at various stages of metabolism.^5^^,^^30^ LC-MS/MS lipidomics of plasma samples opens a more prolific view on circulating lipids (Figure 1K-N and Table 2). Even so, it is an elementary biological outcome that only a few lipid classes account for most of the circulating lipids. The core of circulating lipoprotein particles consists principally of TG and CE, comprising around 60% of circulating lipids, while phosphatidylcholines and FC—key molecular components of lipoprotein surfaces—comprise around 25% of circulating lipids. Free fatty acids and sphingomyelins amount to some 3% each, and lysophosphatidylcholines as well as diglycerides to around 1% of circulating lipids. The other molecular lipid classes contribute less than 1% per class.

In the case of epidemiologic studies of serum/plasma, based on the fundamental molecular framework illustrated in Figure 1, it is apparent that the lipoprotein measures inevitably affect almost all the lipidomics data associations. In the following, we will present novel data on the molecular associations of lipoprotein and lipidomics measures and aim to reveal the most important lipoprotein attributes that influence the lipidomics associations as well as to provide guidelines for adjustments in epidemiologic studies.

### Molecular clusters and associations within the lipoprotein and lipidomics data

Figure 2 illustrates how the 9 key lipoprotein measures associate with the 24 lipidomics lipid classes. The general association characteristics are summarized via 4 and 8 metabolic clusters for the lipoprotein and lipidomics data, respectively. The lipoprotein data clusters combine circulating TG and VLDL-C (cluster 1), apoB and remnant-C (cluster 2), HDL-C and apoA-I (cluster 3), and total, LDL, and IDL cholesterol (cluster 4). These clusters are as expected based on the interrelationships of these lipoprotein measures, as depicted in Figure 1F. Fundamentally, for example, LDL-C is roughly 50% of all circulating cholesterol, and remnant particles make up approximately 30% of apoB-containing lipoprotein particles and carry around 80% of TG in the bloodstream.^5^ The latter fact is behind the key differences in the association behavior of remnant-C and LDL-C, particularly with HDL-C and apoA-I.

The association and clustering analyses shown in Figure 2 for the lipoprotein and lipidomics data are novel. Three clusters for the lipid classes relate to over 90% of the circulating lipid concentrations: clusters 1 (30.5%), 3 (15.2%), and 8 (45.0%). Cluster 1 consists of 5 lipid classes: 2 for oxidized lipids, phosphatidylcholines, free cholesterol, and ceramides. Cluster 3 is di- and triglycerides, and cluster 8 is sphingomyelins and cholesteryl esters. All these clusters associate strongly and positively with the lipoprotein cholesterol cluster 4. Notably, FC and CE measures from lipidomics fundamentally make up the circulating TC and thereby represent key parts of LDL-C as well as other apoB-containing lipoprotein particles. In a similar fashion, the lipidomics TG plus DG represent an independently measured circulating lipoprotein TG, thus having a very high positive correlation between them (lipidomics cluster 3) with lipoprotein cluster 1 as well as a negative correlation with HDL-C.

The association behavior of ceramides, as part of lipidomics cluster 1, is important to note in relation to the recent interpretations suggesting an independent role for ceramides (and phosphatidylcholines) as biomarkers for cardiovascular disease risk.^48^^-^^50^ The (lipoprotein) independent role of ceramides (and phosphatidylcholines) comes into question since they strongly and positively associate with all 7 non-HDL-related lipoprotein measures (Figure 2), all causal for coronary heart disease.^11^

Free fatty acids form the fourth most abundant lipidomics cluster (4.3%). Apart from a very weak association with HDL-C, they do not associate with any lipoprotein measure. The 4 other lipidomics clusters represent markedly rarer lipid classes than the aforementioned 4 (see supplementary material, Extended Results and Discussion).

The associations between lipoprotein and lipidomics data have not been analyzed at this level of detail before. Nevertheless, while these associations (as displayed in Figure 2) are presented explicitly for the first time, they are mostly what could be foreseen based on the fundamental molecular context (Figure 1). These data and findings demonstrate excellent methodological and epidemiologic consistency and confirm the compatibility of the LC-MS/MS lipidomics and NMR spectroscopy platforms at large-scale population studies. In the following 2 sections, we will demonstrate that the pivotal epidemiologic message of these associations is in the correlation structure and in the nonuniform overlap of the various lipoprotein measures.

### Lipoprotein adjustments substantially modify associations between the lipoprotein and lipidomics data

Figure 3 demonstrates how the adjustments for the lipoprotein measures and their combinations impact the associations between the 9 key lipoprotein measures and the 24 lipidomics lipid classes (numerical values are given in Tables S2-S4). Figure 3A reiterates the base results for the associations from Figure 2 (for the convenience of direct comparisons). Individual adjustments for each of the lipoprotein lipids result in marked and characteristic changes in the association patterns (Figure 3B-E). Overall, the effects of these adjustments are logical. Adjusting for TC (Figure 3C) diminishes (or inverts) associations for those lipid classes that are strongly associated with various lipoprotein cholesterol measures (Figure 3A), and adjusting for TG (Figure 3B) does have the strongest effects on the di- and triglyceride associations in the lipidomics data. The effects for adjusting for HDL-C are rather limited (Figure 3E) and those for LDL-C (Figure 3D) similar to those for TC (Figure 3C).

Adjusting for the combination of the standard clinical lipid measures (TG + TC + HDL-C) results in pronounced weakening of all the associations (Figure 3F). Adjustment for TG + LDL-C – HDL-C also results in markedly weakened associations (Figure 3G). Adjusting for apoB (Figure 3H), apoA-I (Figure 3I), and both (Figure 3J) results in another type of prominent changes in the associations. The effects of apoB adjustment show characteristics from adjusting for TG as well as for TC, which is logical since the apoB-particles are the main transporters of both triglycerides and cholesterol in the circulation. Please see supplementary material, Extended Results and Discussion, for further details.

It would be straightforward to take the coherent depletion of the associations between the lipoprotein and lipidomics measures, as a result of the combined TG + TC + HDL-C (or TG + LDL-C – HDL-C) adjustment, as an indication of an efficient and recommendable way to adjust in lipidomics studies. However, as we will exemplify in the following, this is not the case.

### Combined lipoprotein adjustments can unsystematically bias the lipidomics associations

Figure 4 shows that almost all the circulating lipid class concentrations associate with BMI if the model is adjusted only for sex (+ age in YFS). These results are in overall correspondence with those recently reported.^23^ However, the detailed effects of lipoprotein metabolism on these associations have not been previously analyzed in detail. The focus here is solely on these effects as detailed above.

The following discussion depicts the key extreme examples of the effects of lipoprotein adjustments on the outcome associations of the lipidomics measures:

- *Adjusting for an individual lipoprotein measure nullifies the association, but combined lipoprotein lipid adjustments show robust associations.*

The association of sphingomyelins (SM; lipidomics cluster 8 in Figure 4) with BMI is a good example for the generation of robust positive results due to the combined lipoprotein adjustments (TG + TC + HDL-C and TG + LDL-C + HDL-C). Instead, individual adjustments for TG and LDL-C abolish the association. Also, the apolipoprotein adjustments have strong effects on the association, implying a different inherent association structure between lipoprotein lipid and apolipoprotein measures (Figure 1F and Supplementary Figure 6, which shows the associations of the lipoprotein measures with BMI). A more detailed metabolic interpretation of the SM associations is given in the supplementary material, Extended Results and Discussion.

The association of alkenylphosphatidylethanolamines (PE(P); lipidomics cluster 4 in Figure 4) is also a representative example of an adjustment leading to positive results. There is neither a baseline association for PE(P) with BMI nor any of the individual TG, TC, or LDL-C adjustments. Nevertheless, the combined lipoprotein adjustments lead to robust positive associations for PE(P) with BMI. While with the observational data, the correct answer is obscured, if a lipoprotein measure exists that alone abolishes the lipid association, it would suggest that if a combination that includes the same lipoprotein measure creates an association, that association is likely incorrect.

- *Combined lipoprotein lipid adjustments nullify the association, but adjustments with individual lipoprotein measures show robust associations.*

The association of phosphatidylinositols (PI; lipidomics cluster 2 in Figure 4) with BMI is a good example for the generation of negative results due to the combined lipoprotein adjustments. The baseline association and those adjusted individually for the lipoprotein measures show a robust association for PI with BMI. However, the combined lipoprotein adjustments abolish the association.

The above examples pinpoint the most obvious challenges to combined lipoprotein lipid adjustments in interpreting epidemiologic associations of lipidomics measures—the combined adjustments can, inconsistently, result in robust associations when already one lipoprotein measure explains the association and nullify an association that appears robust for adjustments with all the individual lipoprotein measures. Figure 4 also illustrates various more subtle situations in which the meaning and comparison of the association effects are obscured. This is the first time that these effects have been identified and compared in detail, although they might not come as a surprise in an epidemiologic context.^11^^,^^25^^,^^26^^,^^45^^,^^51^

In addition to the meta-analyses for both cohorts and the adjustments with the 9-measure clinical lipoprotein panel (Figure 4), we also assessed the effects of adjusting for lipoprotein(a) (Lp(a)) for the associations of the 24 most abundant lipidomics lipid classes with BMI in YFS, as illustrated in Figure S7 (Lp(a) measurements are not available in NFBC66). Lp(a) is typically not involved in the clinical lipoprotein measurements since its circulating concentration is very low in most individuals. Lp(a) concentration is also known to be independent of the main lipoprotein concentrations.^32^ In concordance with these, the Lp(a) adjustments had no effect on the associations of the lipidomics lipid classes with BMI. Thus, only if a notable portion of the entire pool of a circulating lipid is mediated via certain lipoprotein particles, the corresponding adjustments will have strong effects on the associations. This is further exemplified by the results with simulated data in the next paragraph.

### Simulated data for lipoprotein mediation and nonmediation of circulating lipids

The simulated mediation scenarios depicted in Figure 5 (ie, pure mediation and no mediation by lipoprotein particles) clearly illustrate the complexity introduced by the highly correlated lipoprotein metabolism to the associations of their lipid constituents. For the simulated lipid 1, we did not introduce any direct association between the lipid and BMI. However, the results show that the lipoprotein mediation per se can create a positive association. In addition, the lipoprotein adjustment can either enhance (apoA-I) or reverse (apoB) the (nonexistent) association. It is (correctly) nullified by the TG adjustment. In a real observational situation, there is no way of knowing which one would be correct.

Overall, we wish to emphasize that the simulation is a pure linear mediation model while the lipoprotein system is more complex with nonlinear bidirectional causality (Figure 5A). As we do not know the true causal structure in detail, it is unfeasible to provide exact recipes for how to handle the complexity when choosing adjustments. Observational data are also affected by the likely presence of unknown epidemiologic confounding. Nevertheless, the simulated data with the marked differences between the lipoprotein adjustments for the lipoprotein-mediated and nonmediated circulating lipid (Figure 5B) complement the observational findings and concur with the key role of lipoprotein mediation in the interpretation of lipidomics results. This calls for multiple types of adjustments and case-by-case interpretation based on metabolic characteristics of the adjusting measures.

## Conclusions

These analyses demonstrate that the fundamental lipoprotein-centered transport of circulating lipids is such a complex and intertwined molecular system that it casts doubt on any “lipoprotein-independent” interpretation of lipidomics results and even for the search for such interpretations. These results call for awareness of the inherent structural and mediating role of lipoprotein particles in systemic lipid metabolism. Incorporation of lipoprotein data in an appropriate manner is crucial for correct analyses and interpretations of epidemiologic (and genetic) lipidomics data. The combined lipoprotein adjustments are those that accentuate the inherent complexities. It would be more sensible to perform adjustments separately with a few key individual lipoprotein measures (eg, TG, LDL-C, HDL-C, and apoB). Albeit often still strenuous, the effects of these rather specific adjustments can, at least in principle, be interpreted based on the known compositional characteristics of lipoprotein particles and their overall roles in lipoprotein metabolism.

## Supplementary material

Supplementary material is available at *American Journal of Epidemiology* online.

## Disclaimer

The study funders were not involved in the design of the study; the collection, analysis, and interpretation of data; writing the report; or imposing any restrictions regarding the publication of the report.

## Supplementary Material

Web_Material_kwae445

## Data Availability

The data sets used in the current study are available from the cohorts through application process for researchers who meet the criteria for access to confidential data: https://www.oulu.fi/nfbc/ (NFBC66) and http://youngfinnsstudy.utu.fi (YFS). Regarding the YFS data, the Ethics Committee has concluded that under applicable law, the data from this study cannot be stored in public repositories or otherwise made publicly available. The data controller may permit access on a case-by-case basis for scientific research, not however to individual participant level data, but aggregated statistical data, which cannot be traced back to the individual participants’ data.
