# Supplementary material for: Understanding lipidomics associations and the lipoprotein-related caveats in population epidemiology
Source: Am J Epidemiol. 2024 Nov 28;194(10):2800–12. doi: 10.1093/aje/kwae445 (PMC12527231; doi:10.1093/aje/kwae445)
Supplement: Web_Material_kwae445 [file web_material_kwae445.zip › AJE Supplementary material REVISION_FINAL.pdf]

Understanding lipidomics associations and the lipoprotein-related caveats in population epidemiology

Siyu Zhao, Pauli Ohukainen, Johannes Kettunen, Marjo-Riitta Järvelin, Mika Kähönen, Terho Lehtimäki, Jorma Viikari, Olli T. Raitakari, Ville-Petteri Mäkinen, Mika Ala-Korpela

SUPPLEMENTARY MATERIAL

Study populations

Northern Finland Birth Cohort 1966

Cardiovascular Risk in Young Finns Study

LC-MS/MS mass spectrometry analysis for the molecular lipid classes

Extended results and discussion

Molecular clusters and associations within the lipoprotein and lipidomics data

Lipoprotein adjustments substantially modify associations between the lipoprotein and lipidomics data

Combined lipoprotein adjustments can unsystematically bias the lipidomics associations

References

Supplement Tables

Table S1. The number of outliers in each measure in NFBC66 and in YFS.

Table S2. (Excel) Partial Spearman's rank correlations for the associations of the nine key lipoprotein measures with the 24 most abundant lipidomics lipid classes adjusted for different lipoprotein measures and their combinations and meta-analysed for both cohorts (**Figure 2** and **Figure 3**).

Table S3. (Excel) Partial Spearman's rank correlations for the associations of the nine key lipoprotein measures with the 24 most abundant lipidomics lipid classes adjusted for different lipoprotein measures and their combinations. Cohort specific results in NFBC66. (**Figure S2**).

Table S4. (Excel) Partial Spearman's rank correlations for the associations of the nine key lipoprotein measures with the 24 most abundant lipidomics lipid classes adjusted for different lipoprotein measures and their combinations. Cohort specific results in YFS. (**Figure S3**).

Supplement Figures

Figure S1. The key compositional, circulatory, and association characteristics of the 9-measure lipoprotein panel for (A) NFBC66 and (B) YFS.

Figure S2. Associations of the nine key lipoprotein measures with the 24 most abundant lipidomics lipid classes as indicated by Partial Spearman's rank correlations and adjusted for different lipoprotein measures and their combinations. Cohort specific results in NFBC66.

Figure S3. Associations of the nine key lipoprotein measures with the 24 most abundant lipidomics lipid classes as indicated by Partial Spearman's rank correlations and adjusted for different lipoprotein measures and their combinations. Cohort specific results in YFS.

Figure S4. The effects of various lipoprotein adjustments on the associations of the 24 most abundant lipidomics lipid classes with BMI via linear regression analyses in NFBC66.

Figure S5. The effects of various lipoprotein adjustments on the associations of the 24 most abundant lipidomics lipid classes with BMI via linear regression analyses in YFS.

Figure S6. The associations of the key lipoprotein measures with BMI with various adjustments. (A) Meta-analysis, (B) NFBC66, and (C) YFS.

Figure S7. The effects of the Lp(a) adjustment on the associations of the 24 most abundant lipidomics lipid classes with BMI via linear regression analyses in YFS.

Study populations

Northern Finland Birth Cohort 1966

The Northern Finland Birth Cohort (NFBC) studies are two longitudinal birth cohorts established to study factors affecting preterm birth and consequent morbidity in the two northernmost provinces of Finland, Oulu, and Lapland. The NFBC66 includes 12,058 live births (12,231 children) covering 96% of all eligible births in this region during January – December 1966. Data and sample collection was conducted in 2012 at the age of 46 years, including clinical examination and questionnaires.[1] The serum samples (n=5,657) in this study used for the NMR spectroscopy and the LC-MS/MS analyses were taken after overnight fasting. The research protocols were approved by the Ethics Committee of University of Oulu and the Ethics Committee of Northern Ostrobothnia Hospital District, Finland. More information on the cohort and the data collection can be found at http://www.oulu.fi/nfbc.

Cardiovascular Risk in Young Finns Study

The Cardiovascular Risk in Young Finns Study (YFS) is a population based prospective cohort study conducted with the aim of studying the levels of cardiovascular risk factors in children and adolescents in different parts of the country.[2] The first cross-sectional survey was conducted in 1980. Total sample size was 4,320 boys and girls in 6 age cohorts (aged 3, 6, 9, 12, 15 and 18); these subjects were randomly chosen from the national register. After that, several follow-up studies of this cohort have been conducted. Examinations have included comprehensive data collection using questionnaires, physical measurements, and blood tests. The follow-up during 2011-2012 used in this study comprise 2,036 subjects. All serum samples were taken after overnight fasting. All participants gave written informed consent, and the study was approved by the local ethics committees and conducted in accordance with the Declaration of Helsinki. More information on the cohort can be found at https://youngfinnsstudy.utu.fi/index.html.

LC-MS/MS mass spectrometry analysis for the molecular lipid classes

The lipid analyses were performed using triple quadruple mass spectrometer (Agilent 6490 QQQ) with an Agilent 1290 series HPLC system and a ZORBAX eclipse plus C18 column (2.1 × 100 mm × 1.8 µm). Solvent A consisted of 50% H_2O/ 30% acetonitrile / 20% isopropanol with 10mM ammonium formate and 5µM medronic acid, while solvent B consisted of 1% H_2O/ 9% acetonitrile / 90% isopropanol with 10mM ammonium formate. Mass spectrometry analysis was performed in a positive ion mode with dynamic scheduled multiple reaction monitoring (MRM). We modified the methodology to include a dual column setup (in which one of the columns is set to equilibrate while the other is running a sample). The temperature within the column compartment was set to 45 °C with the chromatographic conditions as follows: starting at 15% solvent B and increasing to 50% B over 2.5 min, then quickly ramping to 57% B for 0.1 min. For 6.4 min, %B was increased to 70%, then increased to 93% over 0.1 min and increased to 96% over 1.9 min. The gradient was quickly ramped up to 100% B for 0.1 min and held at 100% B for a further 0.9 min. This was a total run time of 12 min. The column was then brought back down to 15% B for 0.2 min and held for another 0.7 min prior to switching to the alternate column for running the next sample. The column that was being equilibrated was run as follows: 0.9 min of 15% B, 0.1 min increase to 100% B and held for 5 min, decreasing back to 15% B over 0.1 min and held until it was switched for the next sample. We used a 1 µL injection per sample and the following mass spectrometer conditions were used: gas temperature, 150 °C; gas flow rate, 17 L/min; nebulizer, 20 psi; sheath gas temperature, 200 °C; capillary voltage, 3500 V and sheath gas flow, 10 L/min.

Extended results and discussion

Molecular clusters and associations within the lipoprotein and lipidomics data

The associations for cluster no. 2 (accounting for 1,1% of circulating lipid concentration) are similar to those for cluster no. 1. Cluster no. 5 (1,9% of circulating lipids) and cluster no. 6 (0,1% of circulating lipids) both have rather weak associations with the lipoprotein measures, but the association profiles are rather similar to those of lipidomics cluster no. 8. Six lipid classes form cluster no. 4 that accounts for 1.8% of circulating lipids (**Figure 2**). Their associations are similar to those of clusters no. 5, 6, and 8 with respect to the lipoprotein clusters no. 2, 3, and

4. However, their associations with lipoprotein cluster no. 1 are inconsistent and weak, except the negative associations of dehydroxycholesterol esters with TG and VLDL-C.

Lipoprotein adjustments substantially modify associations between the lipoprotein and lipidomics data

Based on the fundamental molecular framework (**Figure 1**) and the correlation results illustrated in **Figure 2** and **3**, it is not unexpected that we see large effects in the associations of the circulating lipid class concentrations with BMI due to various lipoprotein adjustments (**Figure 4**). The TG adjustment affects strongly on multiple lipid classes, especially in clusters no. 1, 2 and 3 (and DE in cluster no. 4). Adjusting for HDL-C also has a strong effect on a few lipid classes (even though the effects of HDL-C adjustment for the correlations between the lipoprotein panel and the lipidomics measures are mostly not that pronounced; **Figure 3E**). Particularly lipid classes forming clusters no. 3 and no. 4 are strongly affected by the adjustment for HDL-C. Overall, adjusting for TC has only minor or no effects on the lipidomics associations with BMI.

The effects of adjusting for the combinations of the clinical lipid measures (TG+TC+HDL-C or TG+LDL-C+HDL-C) are almost identical and strong for multiple lipid classes. Similarly to the effects of adjusting for TG and HDL-C, the adjustment effects for apoB and apoA-I are often to opposite directions as expected based on their metabolic roles. Notably, adjusting for apoA-I abolished the association between cholesteryl esters and BMI. The combined adjustment for apoB and apoA-I is typically a varied combination of the individual apoB and apoA-I adjustments.

Additionally, even though TC has a minor role for the associations between BMI and the lipidomics measures, it would be expected that it might have a more prominent role for some other outcomes, for example, cardiometabolic diseases.

Combined lipoprotein adjustments can unsystematically bias the lipidomics associations

In the manuscript the associations of sphingomyelins (SM; lipidomics cluster no. 8 in **Figure 4**) with BMI are noted as an example of a situation in which the individual adjustments for TG and LDL-C abolish the association, but the combined lipoprotein lipid adjustments (TG+TC+HDL-C and TG+LDL-C+HDL-C) result in robust positive associations. *How should we understand and interpret such results?*

Figure 2 shows that SM is positively correlated with all the individual lipoprotein measures. The sex and age adjusted base association of SM with BMI is positive and robust (0.055 SD in SM per SD-BMI, $p < 0.002$; **Figure 4**). Various lipoprotein adjustments result in marked changes in the SM association; for example, adjusting for apoB changes the result to -0.068 SD in SM per SD-BMI ($p < 0.002$) and adjusting for apoA-I to 0.16 SD in SM per SD-BMI ($p < 0.002$). The result for the HDL-C adjustment is similar to apoA-I. These effects are large and rather untypical from an epidemiological perspective and suggest a mediating role for the lipoprotein measures.

Clearly the situation is more complicated than the straightforward correlations in **Figure 2** reflect. For example, a plain interpretation for the individual apoB and apoA-I adjustment would lead to an opposite conclusion on the association between SM and BMI. On the contrary, the combined adjustment for apoB+apoA-I would suggest no association. This is contrasting the results for the adjustment for the combined lipoprotein lipids, TG+TC+HDL-C and TG+LDL-C+HDL-C, that show robust positive associations between SM and BMI. However, as discussed in the manuscript and illustrated in **Figure 1**, these combined lipid adjustments are tangled due to the relations of the individual measures to the entire lipoprotein cascade. For instance, TC includes HDL-C (that comprises approximately 30% of TC) and TG molecules are transported in all lipoprotein particles, however not in a uniform manner (around 87% in apoB-containing particles and 13% in HDL particles; **Figure 1**). Thus, these typical clinical lipoprotein lipid measures give an incomplete and biased view on lipoprotein metabolism and depending on the overall correlation structure between the lipoprotein and lipidomics measures as well as the outcome (i.e., BMI in this case), the results of these adjustments are convoluted and basically unfeasible to foresee and challenging to interpret.

In fact, the apolipoprotein measures are a lot more specific for the lipoprotein metabolism than the abovementioned clinical lipid measures.[3–6] ApoB exists as a single molecule per lipoprotein particle in the entire VLDL-LDL metabolic pathway and apoA-I is almost entirely present only in HDL particles. Therefore, it is safe to say that the association between SM and BMI is strongly affected by both, the apoB-containing particles, i.e., the liver-to-adipose (and other tissues) metabolic pathways as well as the metabolic pathways related to HDL-particles, e.g., the delivery of circulating cholesterol back to the liver and potentially inflammatory pathways.[7] Evidently, it would be metabolically far too simplified to interpret the result from the combined apoB+apoA-I adjustment that there is no association between SM and BMI. In this case the view via the apolipoprotein measures gives metabolic distinction to the situation and elucidates the incorrectness of the combined lipoprotein lipid adjustments, particularly if interpreted simply as representing "lipoprotein-independent" associations of SM with BMI.

It is also important to note that circulating (total) TG is the single strongest measure to associate with BMI – naturally from both NMR (**Figure S6**) and from lipidomics (**Figure 4**) as these measures represent the same molecular entity. This relationship is well known and represents the causal influences of adiposity.[8] The association of LDL-C with BMI is only about 25% of the TG association (**Figure S6**) but also known to be causal,[8] and adjusting for LDL-C abolishes the association between SM and BMI similarly to the TG adjustment.

In addition, it is well known that lipoprotein lipids are heterogeneously distributed among the different lipoprotein particles. This holds for both the composition (**Figure 1G**) and the circulating lipid concentrations (**Figure 1H**).[5] The distribution of circulating SM molecules is similar to that of phospholipids (PL in **Figure 1H**) 7%, 50%, and 43% in VLDL, LDL, and HDL particles, respectively.[9] Thereby the circulating SM concentration is almost equally reflecting the apoB- and HDL-related opposing metabolic pathways. Nevertheless, this does not add much on the interpretation of the various adjustment effects.

The above discussion exemplifies the inherent complexity related to the lipoprotein and lipidomics data in general. In our opinion, the adjustments for apoB and apoA-I, reflecting the role of different metabolic pathways, are of interest and an example of added value by combining lipoprotein and lipidomics data. We would emphasise the strong (causal) relationship between BMI and TG in combination with the results that the association

between SM and BMI is abolished by both the TG and LDL-C adjustment. It is therefore unlikely that the association between SM and BMI would be independent of lipoprotein lipids. Therefore, importantly, if the interpretation of the association between SM and BMI would be done only by relying on the combined clinical lipid adjustment, the inference would be misleading.

References

1. Nordström T, Miettunen J, Auvinen J, et al. Cohort Profile: 46 years of follow-up of the Northern Finland Birth Cohort 1966 (NFBC1966). *Int J Epidemiol* 2022;**50**:1786–1787j.

2. Raitakari OT, Juonala M, Rönnemaa T, et al. Cohort profile: the cardiovascular risk in Young Finns Study. *Int J Epidemiol* 2008;**37**:1220–1226.

3. Pechlaner R, Tsimikas S, Yin X, et al. Very-Low-Density Lipoprotein-Associated Apolipoproteins Predict Cardiovascular Events and Are Lowered by Inhibition of APOC-III. *J Am Coll Cardiol* 2017;**69**:789–800.

4. Clarke R, Von Ende A, Schmidt LE, et al. Apolipoprotein Proteomics for Residual Lipid-Related Risk in Coronary Heart Disease. *Circ Res* 2023;**132**:452–464.

5. Ala-Korpela M, Zhao S, Järvelin M-R, Mäkinen V-P, Ohukainen P. Apt interpretation of comprehensive lipoprotein data in large-scale epidemiology: disclosure of fundamental structural and metabolic relationships. *Int J Epidemiol* 2022;**51**:996–1011.

6. Glavinovic T, Thanassoulis G, Graaf J de, Couture P, Hegele RA, Sniderman AD. Physiological Bases for the Superiority of Apolipoprotein B Over Low-Density Lipoprotein Cholesterol and Non-High-Density Lipoprotein Cholesterol as a Marker of Cardiovascular Risk. *J Am Heart Assoc* 2022;**11**:e025858.

7. Kontush A. HDL and Reverse Remnant-Cholesterol Transport (RRT): Relevance to Cardiovascular Disease. *Trends Mol Med* 2020;**26**:1086–1100.

8. Würtz P, Wang Q, Kangas AJ, et al. Metabolic signatures of adiposity in young adults: Mendelian randomization analysis and effects of weight change. *PLoS Med* 2014;**11**:e1001765.

9. Wiesner P, Leidl K, Boettcher A, Schmitz G, Liebisch G. Lipid profiling of FPLC-separated lipoprotein fractions by electrospray ionization tandem mass spectrometry. *J Lipid Res* 2009;**50**:574–585.

Supplement Tables

Table S1. The number of outliers (third quartile + 8 x interquartile range) in each measure in NFBC66 and in YFS.

	**NFBC66**	**YFS**
Participants	5,657	2,036
**Lipoprotein panel (NMR)**		
TG	2	3
VLDL-C	0	0
APO-B	0	0
Remnant-C	0	0
HDL-C	0	0
APO-A1	0	0
Total-C	0	0
LDL-C	0	0
HDL-C	0	0
**Lipid classes (LC-MS/MS)**		
Ox-PC/LPC	0	0
Ox-CE	23	2
PC	0	0
Free C	0	0
Cer	0	0
PI	0	0
PE	1	2
TG(O)	6	4
DG	15	7
TG	1	3
PE(P)	0	0
PE(O)	0	1
PC(O)	0	0
GM3	0	0
PC(P)	0	0
DE	3	1
LPC	0	0
LPE	1	0
Hex2Cer	0	0
HexCer	0	0
LPC(O)	0	0
FFA	0	0
SM	0	0
CE	0	0

Supplement Figures

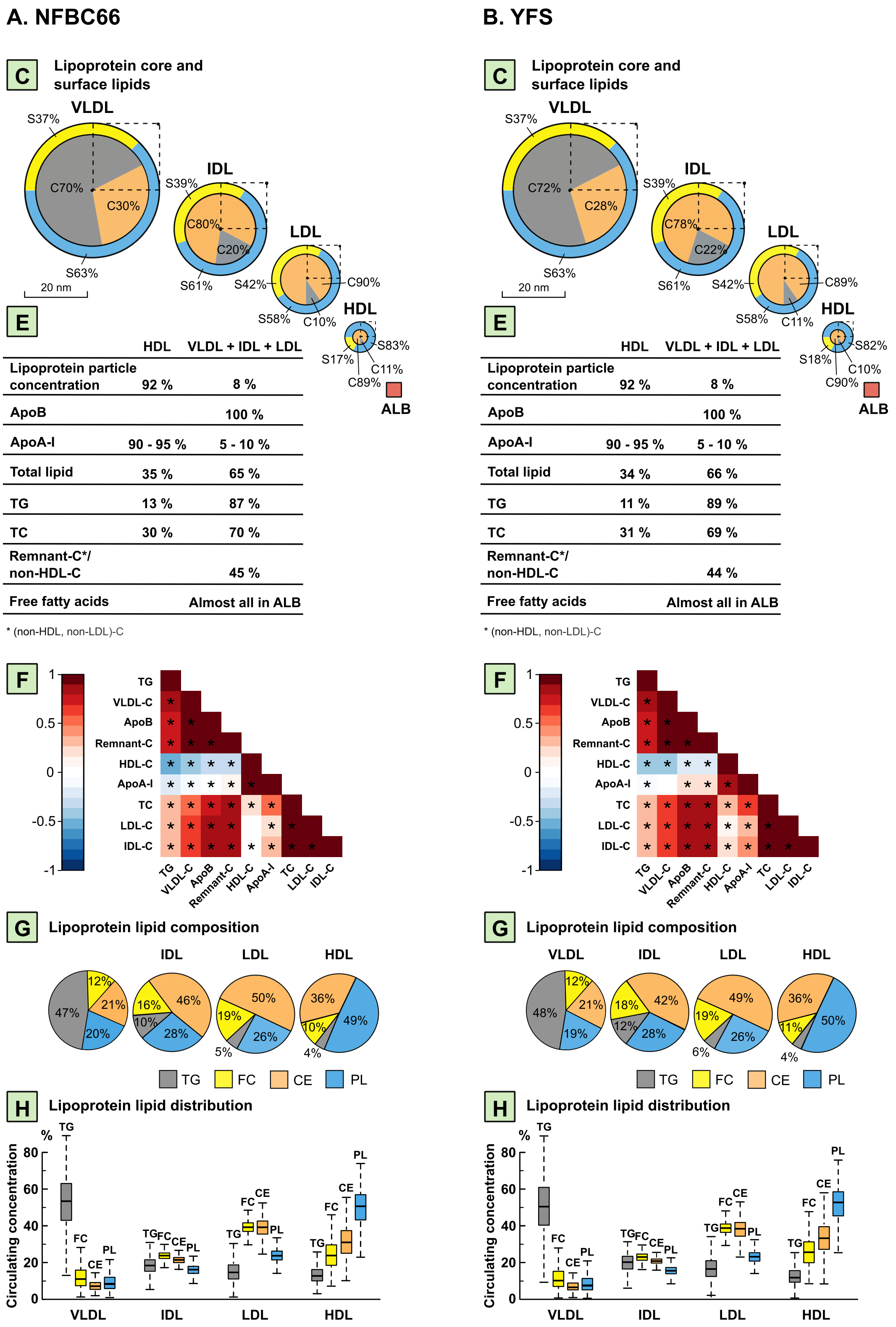

Figure S1. The key compositional, circulatory, and association characteristics of the 9-measure lipoprotein panel for (A) NFBC66 and (B) YFS. Please see details and meta-analysed results in **Figure 1**.

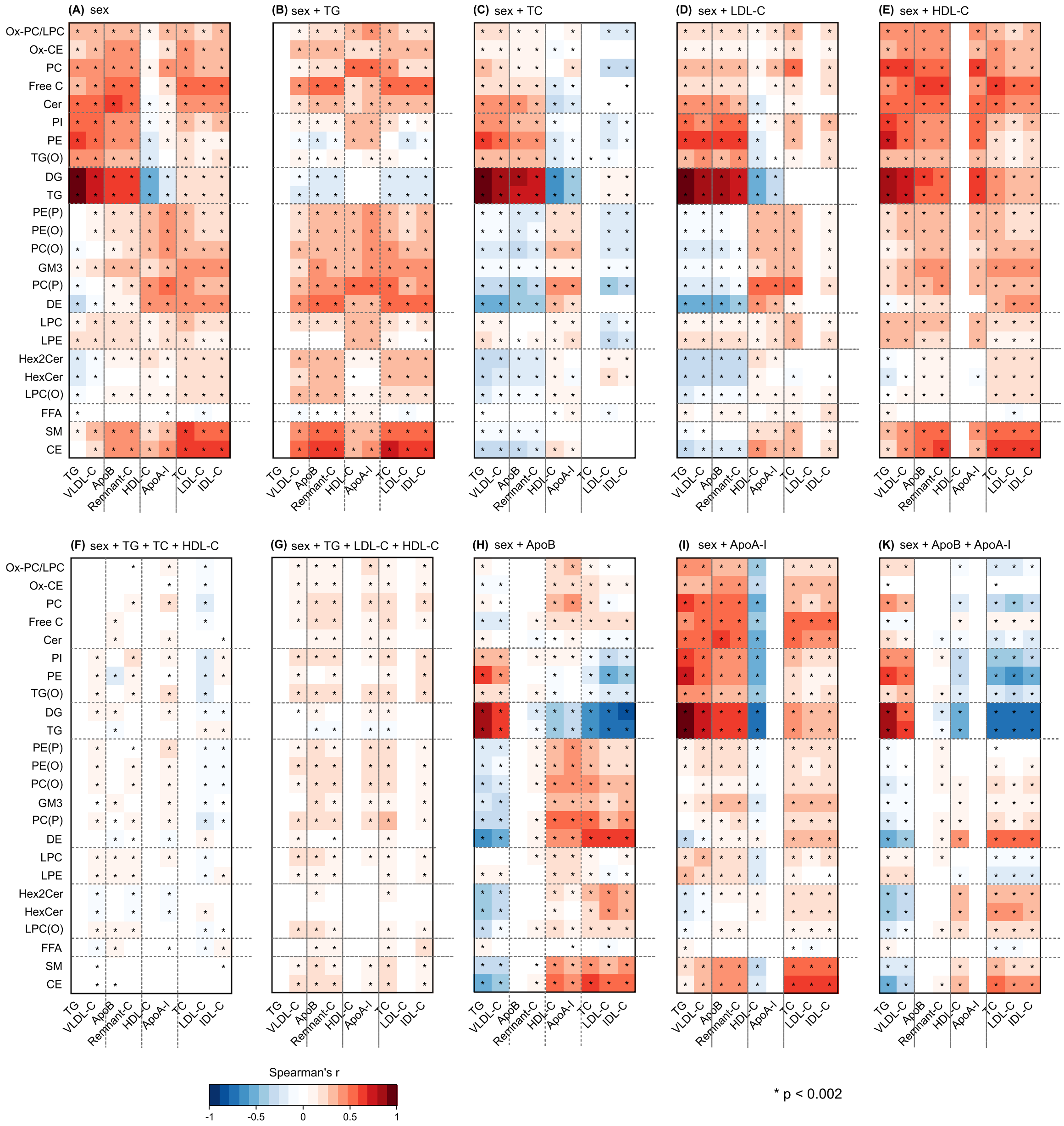

Figure S2. Associations of the nine key lipoprotein measures with the 24 most abundant lipidomics lipid classes as indicated by partial Spearman's rank correlations and adjusted for different lipoprotein measures and their combinations. Cohort specific results in NFBC66. Please see details and meta-analysed results in **Figures 2** and **3**.

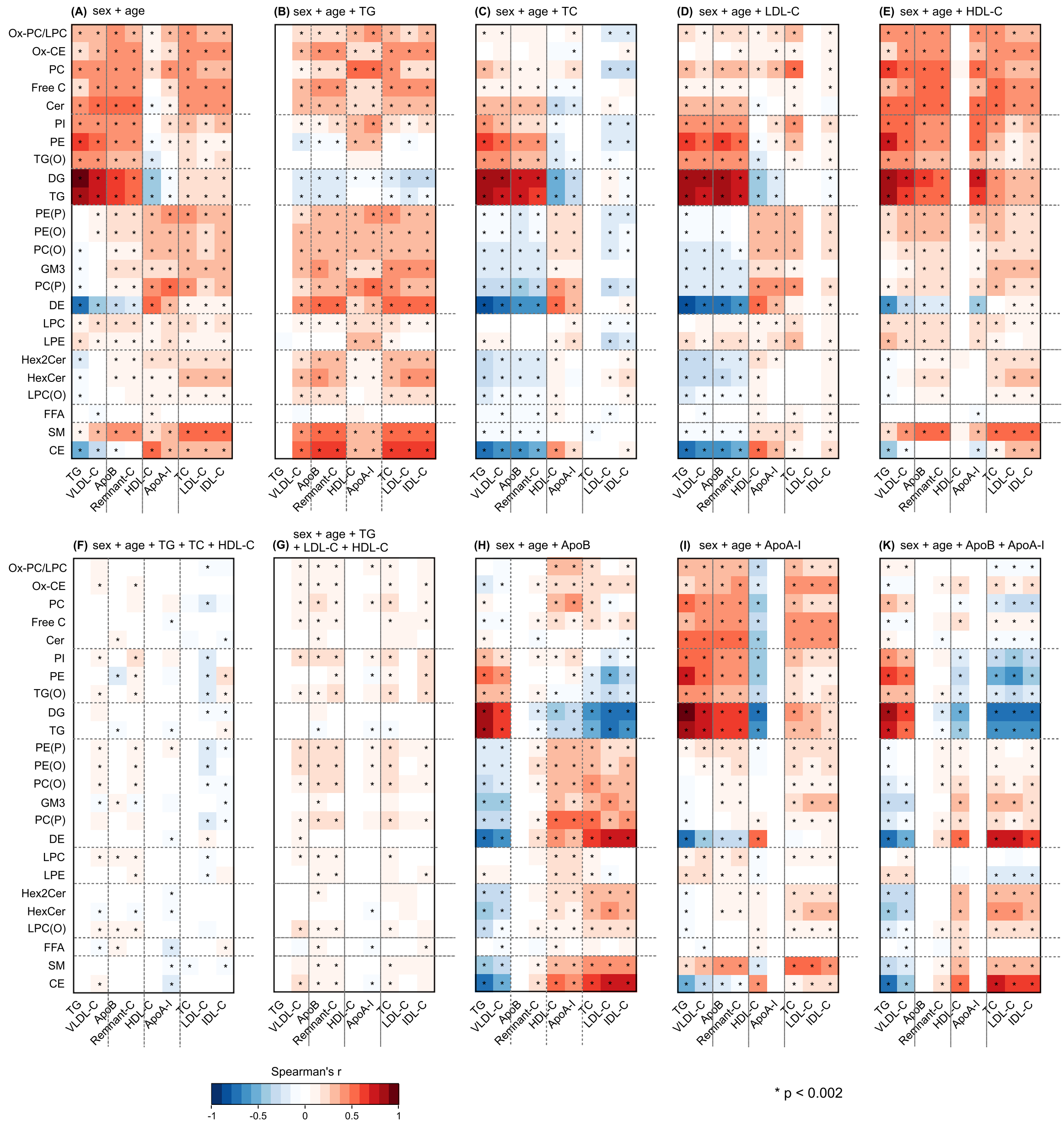

Figure S3. Associations of the nine key lipoprotein measures with the 24 most abundant lipidomics lipid classes as indicated by partial Spearman's rank correlations and adjusted for different lipoprotein measures and their combinations. Cohort specific results in YFS. Please see details and meta-analysed results in **Figures 2** and **3**.

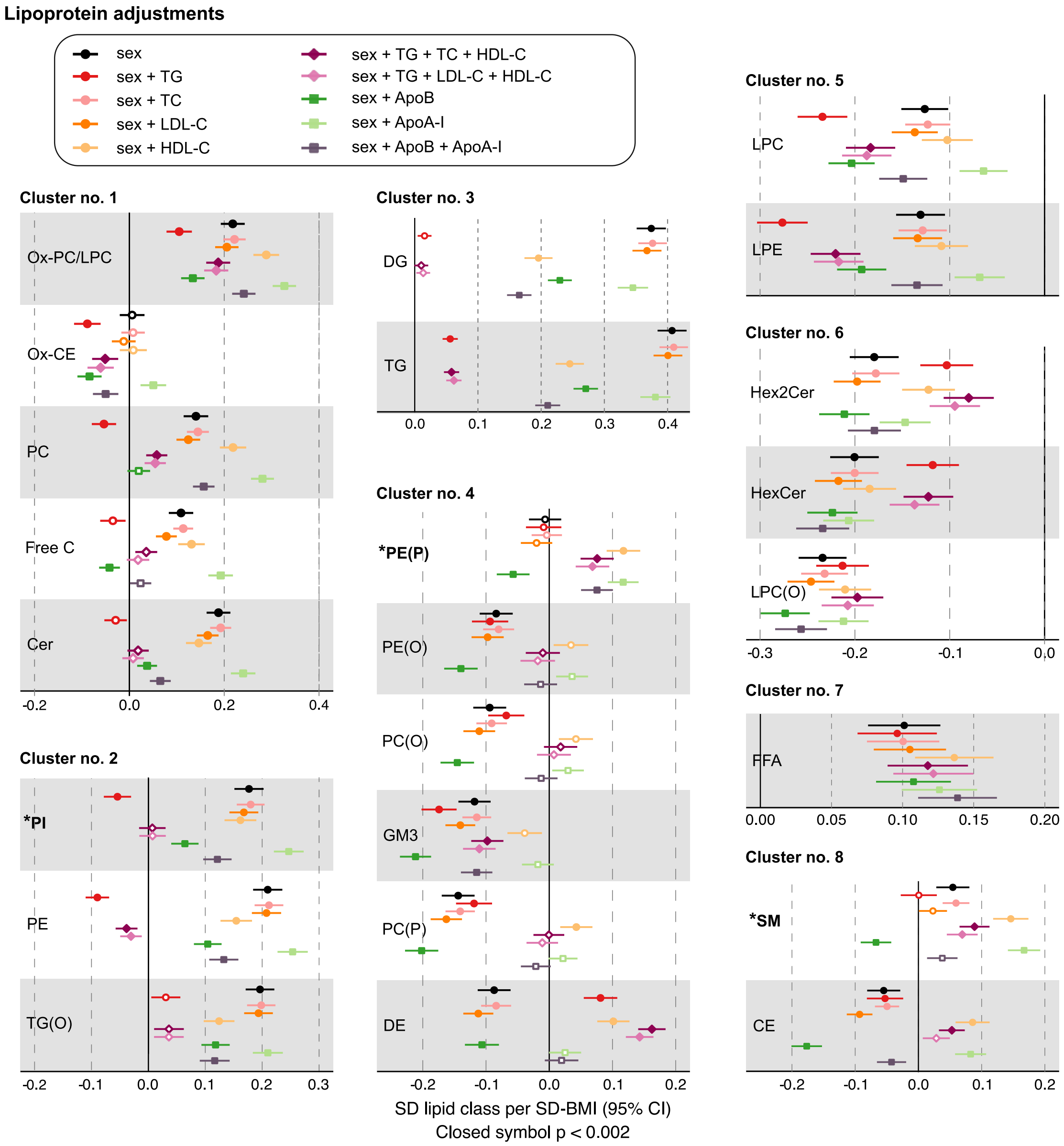

Figure S4. The effects of various lipoprotein adjustments on the associations of the 24 most abundant lipidomics lipid classes with BMI via linear regression analyses in NFBC66. Please see details and meta-analysed results in **Figure 4**.

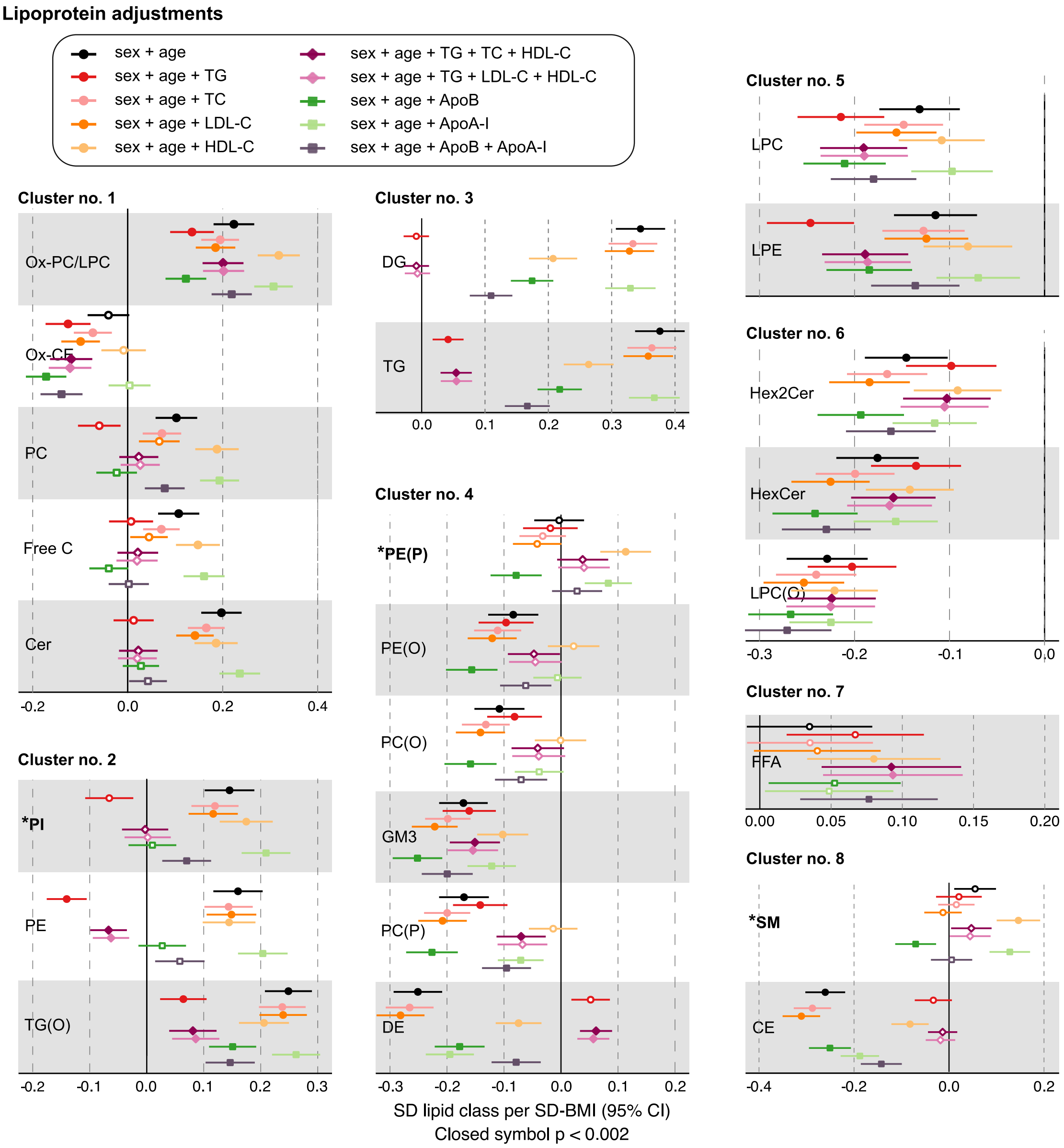

Figure S5. The effects of various lipoprotein adjustments on the associations of the 24 most abundant lipidomics lipid classes with BMI via linear regression analyses in YFS. Please see details and meta-analysed results in **Figure 4**.

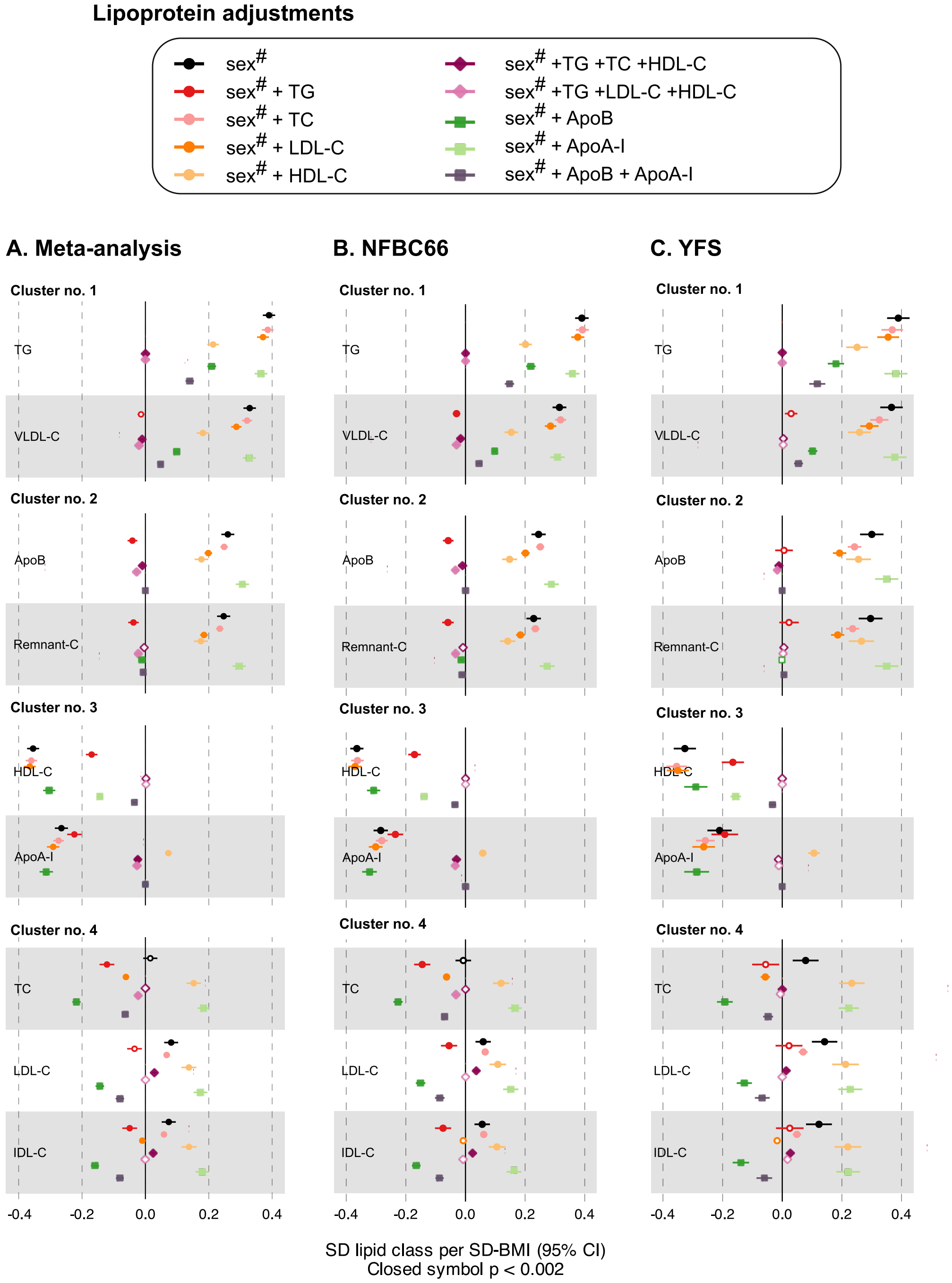

Figure S6. The associations of the key lipoprotein measures with BMI with various adjustments. (A) Meta-analysis, (B) NFBC66, and (C) YFS. Please compare to the lipidomics results in **Figures S4** and **S5**, and in **Figure 4**.

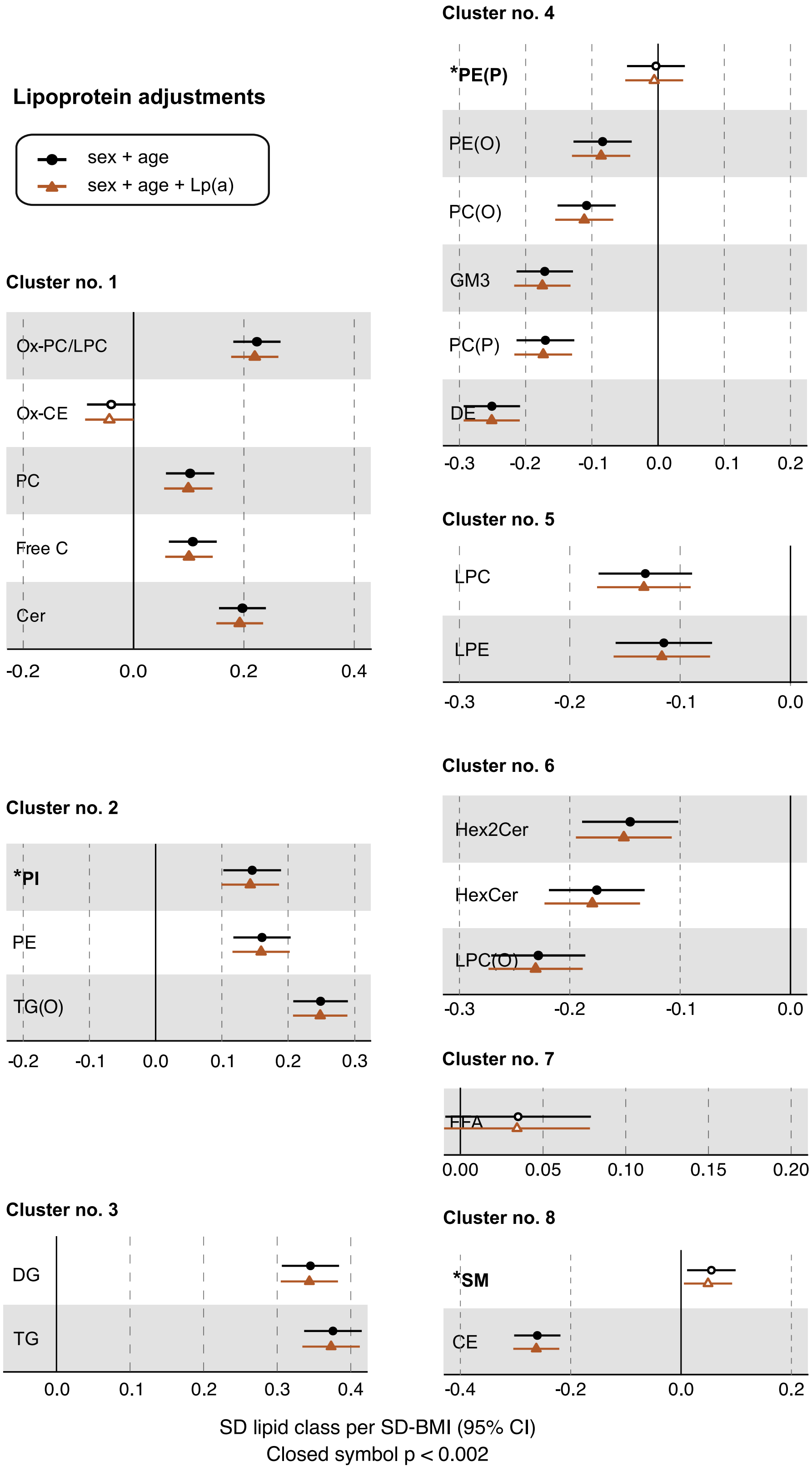

Figure S7. The effects of the Lp(a) adjustment on the associations of the 24 most abundant lipidomics lipid classes with BMI via linear regression analyses in YFS (in which Lp(a) measures were available). There were 2,035 values available for the Lp(a) measurement in YFS.
